# Supplementary material for: Deciphering the Antifibrotic Property of Metformin
Source: Cells. 2022 Dec 16;11(24):4090. doi: 10.3390/cells11244090 (PMC9777391; doi:10.3390/cells11244090)
Supplement: Supplementary file 1 [file cells-11-04090-s001.zip › cells-1990824-supplementary.pdf]

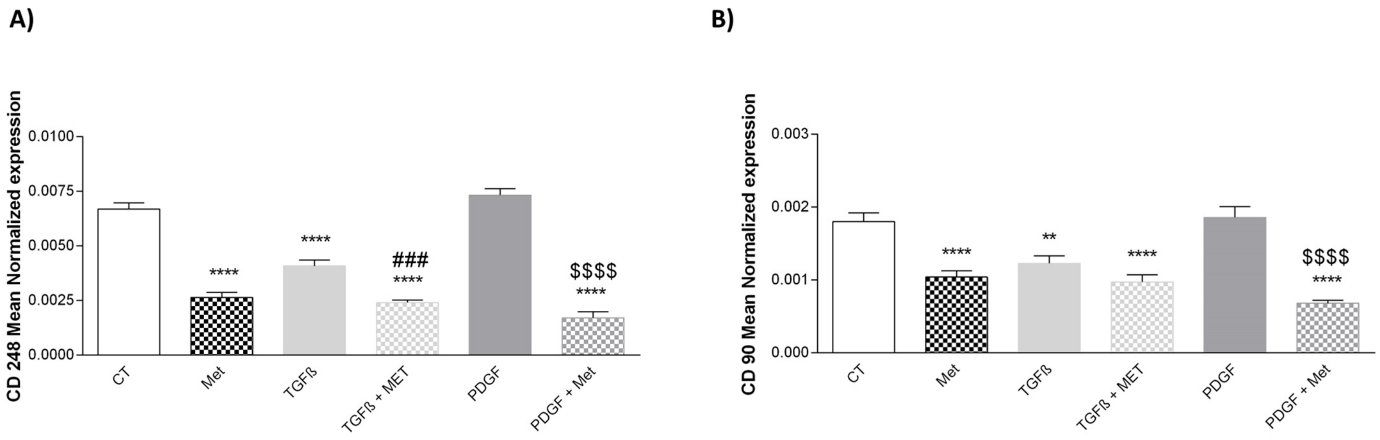

Supplemental Figure 1: Effects of TGF-β1 or PDGF on MSC cell markers (CD90 and CD248). Cells were exposed to TGF-β1 or PDGF at 10ng/mL in association or not with Met (5mM) for 72 h. Then, RNA was collected and (A) CD 248 and (B) CD 90 gene expression levels were determined by qRT-PCR. Reported values are means ± SEM of three independent experiments and p value was calculated using the Bonferroni multiple comparison test: **: $p < 0.01$ and ****: $p < 0.0001$ as compared to control, ###: $p < 0.001$ as compared to TGF-β1 and $$$$: $p < 0.0001$ as compared to PDGF.
